# Factors associated with treatment failure after discharge in inpatients with schizophrenia spectrum disorders: A retrospective observational study

**DOI:** 10.1002/pcn5.70219

**Published:** 2025-10-07

**Authors:** Yoshitaka Saito, Hiroyuki Muraoka, Takayuki Yokoyama, Katsuichiro Shimizu, Yukihiro Tanno, Nagomi Kida, Taiyo Nishikawa, Shin Miyake, Yukio Kenmotsu, Kosei Kamimura, Hirohito Kato, Yuka Noguchi, Seita Tsuchida, Ryutaro Suzuki, Yuki Yoshimura, Shoko Miura, Shunsuke Kamitani, Takaaki Hirooka, Enami Sawayama, Satoru Oishi, Ken Inada

**Affiliations:** ^1^ Department of Psychiatry, Kitasato University School of Medicine Sagamihara Kanagawa Japan; ^2^ Department of Psychiatry Kitasato University Graduate School of Medical Sciences Sagamihara‐shi Kanagawa Japan; ^3^ Department of Psychiatry, Kitasato University, School of Medicine Division of Integrated Psychosocial Care in Community and Child Psychiatry Sagamihara Kanagawa Japan; ^4^ Kitasato University Health Care Center Sagamihara‐shi Kanagawa Japan; ^5^ Kitasato University Kitasato Institute Hospital Minato‐ku Tokyo Japan

**Keywords:** polypharmacy, schizophrenia, treatment failure

## Abstract

**Aim:**

Schizophrenia spectrum disorders are among the most serious mental illnesses that cause social dysfunction. Patients with schizophrenia spectrum disorders are prone to recurrence, and those who require hospitalization are at a high risk; hence, preventing treatment failure after discharge is essential. Therefore, we conducted a retrospective study to identify the factors associated with treatment failure after discharge in patients with schizophrenia spectrum disorders who received psychiatric inpatient treatment.

**Methods:**

This study included 859 patients diagnosed with schizophrenia spectrum and other psychotic disorders who were hospitalized at the Department of Psychiatry in Kitasato University Hospital and Kitasato University East Hospital from January 1, 2014, to December 31, 2021. Treatment failure was defined as discontinuation of outpatient care, psychiatric hospitalization, or death within 1 year after discharge.

**Results:**

Of the 859 patients, 201 (23.4%) experienced treatment failure. Treatment failure rate was 29.0% in patients undergoing antipsychotic polypharmacy, significantly higher than that in patients who were not. Additionally, treatment failure was 20.8% in patients who had a trial overnight stay at home during hospitalization, significantly lower than that in patients who did not.

**Conclusion:**

Antipsychotic polypharmacy at discharge was associated with treatment failure in patients with schizophrenia spectrum disorders. Additionally, trial overnight stays at home during hospitalization potentially contributed to preventing treatment failure. Therefore, preventing treatment failure in patients with schizophrenia spectrum disorder requires optimizing pharmacotherapy and implementing social and environmental adjustments, focusing on the post‐discharge period.

## INTRODUCTION

Schizophrenia spectrum disorders are serious mental illnesses that cause social dysfunction and exhibit a variety of symptoms such as hallucinations, delusions, decreased activity, depression, and cognitive impairment. Schizophrenia is characterized by high rates of relapse,^1^ and hospitalized patients have a high rate of readmission after discharge. Specifically, 13.4% of patients were readmitted within 1 month of discharge, and 38.9% were readmitted within 1 year.^2^ Relapse leads to symptom worsening, cognitive decline, and reduced quality of life.^3^ Therefore, preventing relapse is crucial for treating schizophrenia. Treatment failures resulting from relapses, such as readmission, treatment interruption, and death, are critical clinical outcomes. Identifying and addressing the risk factors is the key to improving the treatment of schizophrenia spectrum disorders.

Pharmacotherapy and psychosocial interventions play important roles in the prevention of relapse in patients with schizophrenia spectrum disorders. Regarding pharmacotherapy, clozapine and long‐acting injections (LAIs) contributed to the prevention of treatment failure in patients with schizophrenia.^4^ Guidelines for schizophrenia recommend monotherapy with antipsychotics; the concomitant use of other psychotropic medications, such as anxiolytics, hypnotics, antidepressants, mood stabilizers, and antiparkinsonian drugs, is not recommended.^5^ However, clinically, antipsychotic polypharmacy is common, and many patients concomitantly use other psychotropic medications.^6^ Antipsychotic polypharmacy is associated with increased risks of adverse events, drug interactions, and tolerability.^7^, ^8^ However, it may prevent treatment failure.^9^ Therefore, further investigation is needed to assess the impact of pharmacotherapy on the prevention of treatment failure in schizophrenia spectrum disorders.

Psychosocial interventions such as family interventions and psychotherapy have proven to be effective.^10^ Although psychoeducation is common for families, psychotherapy is currently scarcely available in Japan because of a lack of resources. Simple social environmental adjustments commonly practiced in Japan for psychiatric inpatients include trial overnight stays at home to assess adaptation to home life during hospitalization and the use of home‐visit nursing after discharge. Whether these commonly implemented measures contribute to preventing treatment failure in patients with schizophrenia spectrum disorders requires further examination. Therefore, in this retrospective study, we aimed to identify the factors associated with treatment failure in patients with schizophrenia spectrum disorder.

## METHODS

### Participants

This study included patients diagnosed with schizophrenia spectrum and other psychotic disorders who were hospitalized at the Department of Psychiatry in Kitasato University Hospital and Kitasato University East Hospital, from January 1, 2014, to December 31, 2021. Diagnoses were classified based on the Diagnostic and Statistical Manual of Mental Disorders, 5th edition (DSM‐5).^11^ An opt‐out procedure was implemented to ensure that patients had the opportunity to refuse participation; those who did not refuse participation were included in the study. Participants who were transferred to another hospital during outpatient visits within the 1‐year evaluation period after discharge were also included. Because this study targeted patients who underwent therapeutic interventions and environmental adjustments during hospitalization, patients with hospitalization durations of 3 days or less were excluded, resulting in a total of 859 participants.

This study was approved by the Ethics Committee of Kitasato University Hospital (approval no. B22‐239, date of approval: June 1, 2023) and conducted in accordance with the Declaration of Helsinki.

### Data collection

We investigated the demographic data (age and sex) of the participants, the percentage of participants who were transferred to another hospital during outpatient visits within the 1‐year evaluation period after discharge, the presence or absence of treatment failure, treatment during hospitalization, and social environmental adjustment. In this study, treatment failure was defined as discontinuation of outpatient care, psychiatric hospitalization, or death within 1 year after discharge, based on a previous study.^9^ Participants who were transferred to another hospital during outpatient visits within the 1‐year evaluation period after discharge were not considered cases of treatment failure. Treatment during hospitalization was evaluated using the following indicators: antipsychotic polypharmacy; modified electroconvulsive therapy (mECT) during hospitalization; and prescription of anxiolytics, hypnotics, antidepressants, mood stabilizers, antiparkinsonian drugs, and LAIs at discharge. For social environmental adjustment, the following were evaluated: trial overnight stay at home during hospitalization, post‐discharge living environment (solitary life or not), and the use of home‐visit nursing after discharge.

### Statistical analysis

Demographic data, the percentage of participants who were transferred to another hospital during outpatient visits within the 1‐year evaluation period after discharge, treatment failure ratio, treatment during hospitalization, and social environmental adjustment were calculated for all participants. Additionally, using the Kaplan–Meier method, we generated a survival curve for the treatment failure ratio during the year following discharge.

Participants were then classified into two groups based on treatment during hospitalization according to the presence or absence of antipsychotic polypharmacy; mECT; and prescription of anxiolytics, hypnotics, antidepressants, mood stabilizers, antiparkinsonian drugs, and LAIs at discharge. The treatment failure ratio was calculated for each group, and the chi‐square test was performed to evaluate the association between treatment during hospitalization and treatment failure.

Additionally, participants were classified into two groups based on their social environmental adjustment, according to post‐discharge living environment (solitary life or not) and the presence or absence of trial overnight stays at home during hospitalization and home‐visit nursing after discharge. The treatment failure ratio was calculated for each group, and a chi‐square test was performed to compare the groups. The significance level of 5% was set at 5.0 × 10^−3^ (0.05/10) by Bonferroni correction for multiple testing with 10 tests.

Survival curves were created using the Kaplan–Meier method for each group with significant differences in the chi‐square tests. In addition, Cox proportional‐hazard regression analysis was performed with treatment failure as the dependent variable. Age, sex, and the items for which significant differences were found in the chi‐square tests were set as independent variables. Participants who were transferred to another hospital during outpatient visits within the 1‐year evaluation period after discharge were treated as censored cases in the Cox proportional‐hazard regression analysis. All statistical analyses were performed using IBM spss Statistics 26.0 (IBM Corp., Armonk, NY, USA).

## RESULTS

**Figure 1:**
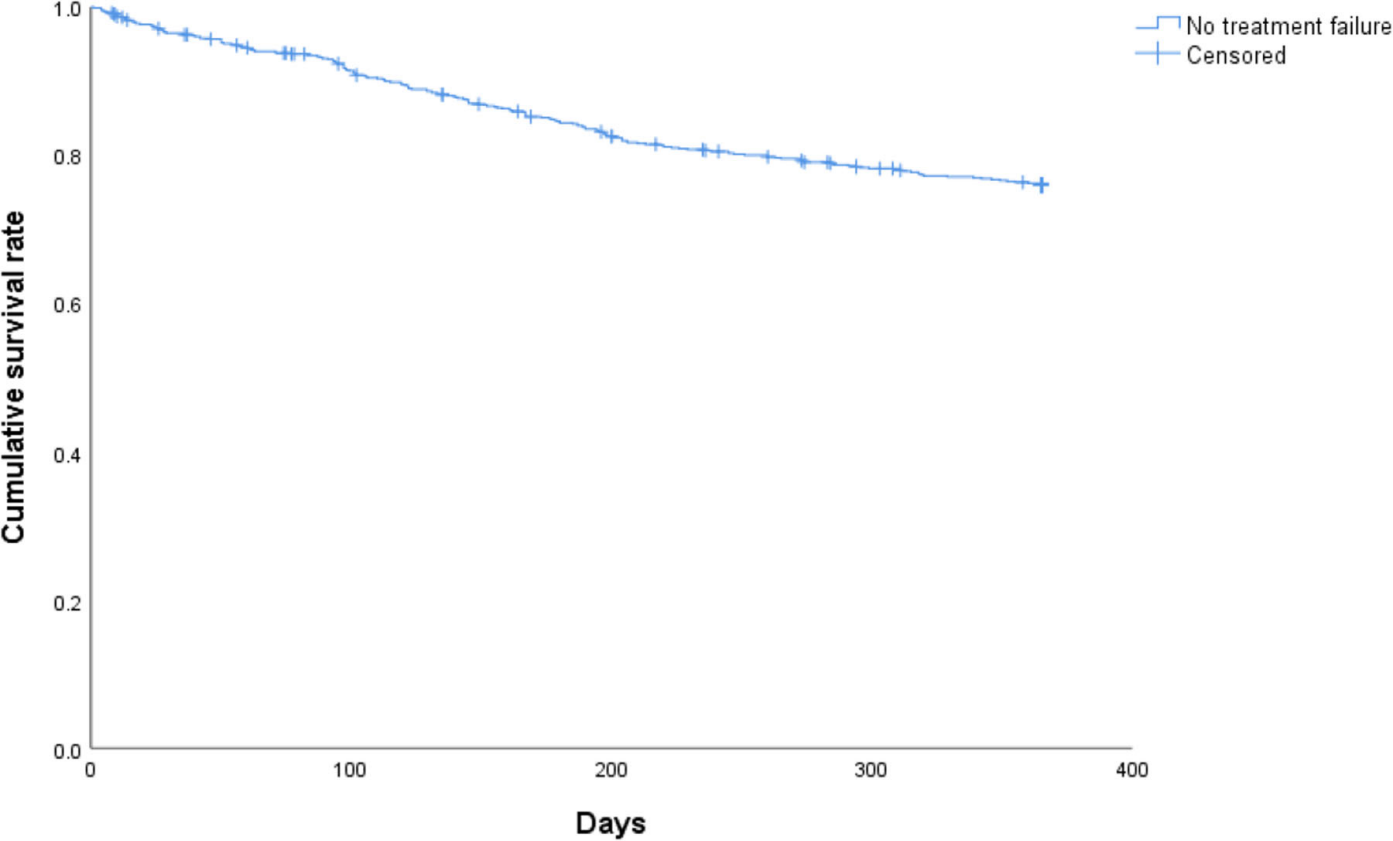
The survival curve for the treatment failure ratio during the first year after discharge. Participants who were transferred to another hospital during outpatient visits within the 1‐year evaluation period after discharge were treated as censored cases.

Demographic data, the percentage of participants who were transferred to another hospital during outpatient visits within the 1‐year evaluation period after discharge, treatment failure ratio, treatment during hospitalization, and social environmental adjustment for all patients are shown in Table 1. Treatment failure occurred in 201 (23.4%) patients. Additionally, 317 (36.9%) patients experienced antipsychotic polypharmacy, and 616 (71.7%) underwent a trial overnight stay at home during hospitalization. Figure 1 shows the survival curve for the treatment failure ratio during the first year after discharge.

**Table 1:** Demographic and clinical data.

	Patients with schizophrenia (*n* = 859)
Age (mean ± SD)	46.10 ± 14.18
Sex (female), *n* (%)	544 (63.3%)
Participants who were transferred to another hospital during outpatient visits within the 1‐year evaluation period after discharge	53 (6.2%)
Treatment failure, *n* (%)	201 (23.4%)
Breakdown of treatment failure	
Discontinuation of outpatient care	23 (11.4%)
Psychiatric hospitalization	173 (86.1%)
Death	5 (2.5%)
Antipsychotic polypharmacy at discharge, *n* (%)	317 (36.9%)
Prescription of anxiolytics and hypnotics at discharge, *n* (%)	412 (48.0%)
Prescription of antidepressants at discharge, *n* (%)	46 (5.4%)
Prescription of mood stabilizers at discharge, *n* (%)	178 (20.7%)
Prescription of antiparkinsonian drugs at discharge, *n* (%)	336 (39.1%)
Using LAIs at discharge, *n* (%)	48 (5.6%)
mECT during hospitalization, *n* (%)	62 (7.2%)
Trial overnight stays at home during hospitalization, *n* (%)	616 (71.7%)
Post‐discharge living environment (solitary life), *n* (%)	127 (14.8%)
Use of home‐visit nursing after discharge, *n* (%)	349 (40.6%)

Abbreviations: LAIs, long‐acting injections; mECT, modified electroconvulsive therapy.

Table 2 shows the relationship between treatment during hospitalization and treatment failure. Treatment failure was 29.0% for patients undergoing antipsychotic polypharmacy at discharge, significantly higher than that for patients who were not (20.1%; *p* = 2.9 × 10^−3^). No significant differences were observed in treatment failure based on the presence or absence of anxiolytics, hypnotics, antidepressants, mood stabilizers, antiparkinsonian drugs, LAIs, or mECT.

**Table 2:** Association between treatment during hospitalization and treatment failure.

Treatment during hospitalization		Treatment failure rate *n* (%)	*χ* ^2^ (df = 1)	*p* value
Antipsychotic polypharmacy at discharge	Yes (*n* = 317)	92 (29.0%)	8.862	*2.9 × 10^−3^
	No (*n* = 542)	109 (20.1%)		
Anxiolytics and hypnotics at discharge	Yes (*n* = 412)	106 (25.7%)	2.396	0.12
	No (*n* = 447)	95 (21.3%)		
Antidepressants at discharge	Yes (*n* = 46)	10 (21.7%)	0.075	0.79
	No (*n* = 813)	191 (23.5%)		
Mood stabilizers at discharge	Yes (*n* = 178)	50 (28.1%)	2.756	0.10
	No (*n* = 681)	151 (22.2%)		
Antiparkinsonian drugs at discharge	Yes (*n* = 336)	78 (23.2%)	0.011	0.92
	No (*n* = 523)	123 (23.5%)		
LAIs at discharge	Yes (*n* = 48)	11 (22.9%)	0.007	0.94
	No (*n* = 811)	190 (23.4%)		
mECT during hospitalization	Yes (*n* = 62)	19 (30.6%)	1.957	0.16
	No (*n* = 797)	615 (22.8%)		

*Note*: As the level of significance, *p* < 5.0 × 10^−3^ was within the 5% significance level, based on the Bonferroni correction, and it was considered in the multiplicity of the tests.

Abbreviations: LAIs, long‐acting injections; mECT, modified electroconvulsive therapy.

* *p* < 0.05, after the Bonferroni correction.

Table 3 shows the relationship between social environmental adjustments and treatment failure. Treatment failure was 20.8% for patients who had trial overnight stays at home during hospitalization, significantly lower than that for patients who did not (30.0%; *p* = 3.9 × 10^−3^). No significant differences were observed regarding treatment failure based on post‐discharge living environment or the presence or absence of home‐visit nursing.

**Table 3:** Association between social environmental adjustment and treatment failure.

Social environmental adjustment		Treatment failure rate *n* (%)	*χ* ^2^ (df = 1)	*p* value
Trial overnight stays at home during hospitalization	Yes (*n* = 616)	128 (20.8%)	8.340	*3.9 × 10^−3^
	No (*n* = 243)	73 (30.0%)		
Post‐discharge living environment (solitary life)	Yes (*n* = 127)	32 (25.2%)	0.269	0.60
	No (*n* = 732)	169 (23.1%)		
Use of home‐visit nursing after discharge	Yes (*n* = 349)	93 (26.6%)	3.460	0.06
	No (*n* = 510)	108 (21.2%)		

*Note*: As the level of significance, *p* < 5.0 × 10^−3^ was within the 5% significance level, based on the Bonferroni correction, and it was considered in the multiplicity of the tests.

* *p* < 0.05, after the Bonferroni correction.

**Figure 2:**
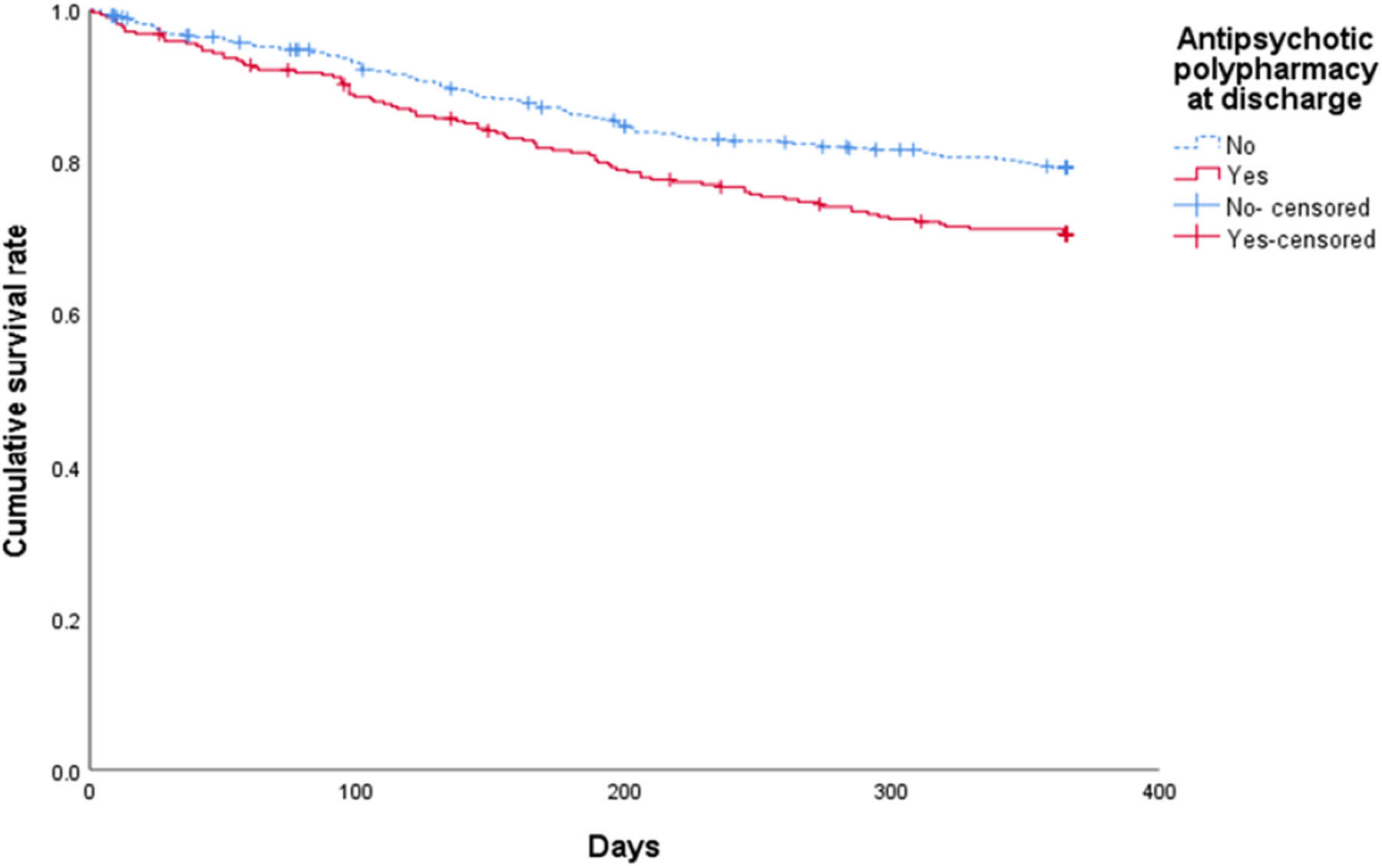
The survival curves for the treatment failure ratio according to the presence or absence of antipsychotic polypharmacy. Participants who were transferred to another hospital during outpatient visits within the 1‐year evaluation period after discharge were treated as censored cases.

**Figure 3:**
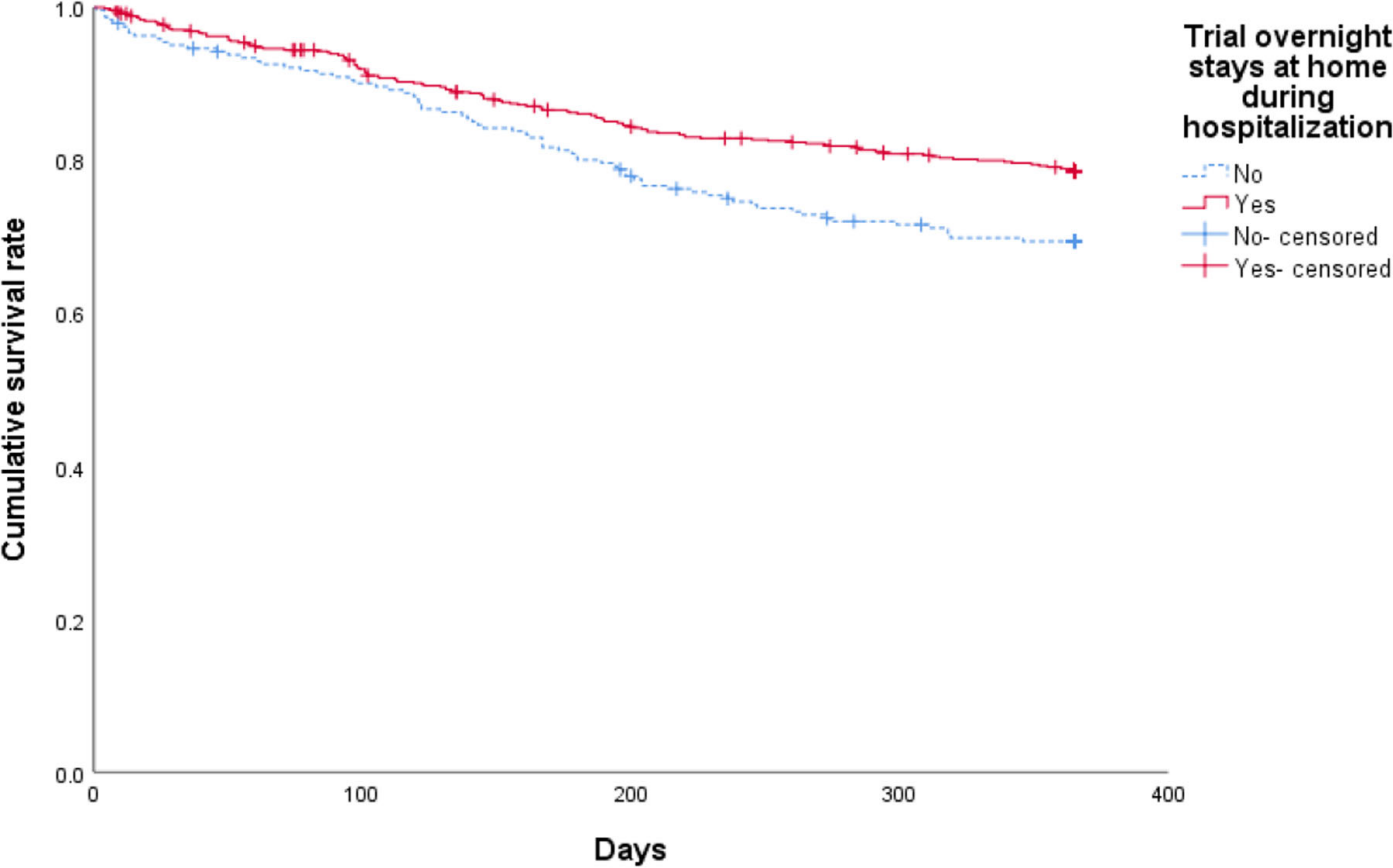
The survival curves for the treatment failure ratio according to the presence or absence of trial overnight stays at home during hospitalization. Participants who were transferred to another hospital during outpatient visits within the 1‐year evaluation period after discharge were treated as censored cases.

Figures 2 and 3 show the survival curves for the treatment failure ratio according to the presence or absence of antipsychotic polypharmacy and trial overnight stays at home during hospitalization. Table 4 shows the results of Cox proportional‐hazard regression analysis with treatment failure as the dependent variable. The Cox proportional‐hazard model shows that antipsychotic polypharmacy at discharge (hazard ratio [HR]: 1.54, 95% confidence interval [CI]: 1.16–2.04) and trial overnight stays at home during hospitalization (HR: 0.68, 95% CI: 0.51–0.91) were significantly associated with treatment failure.

**Table 4:** Results from Cox proportional‐hazard regression analysis for treatment failure.

Independent variables	Hazard ratio	95% confidence interval		*p* value
		Lower limit	Upper limit	
Age	1.01	1.00	1.02	0.09
Sex (female)	0.97	0.73	1.29	0.83
Antipsychotic polypharmacy at discharge	1.54	1.16	2.04	*2.5 × 10^−3^
Trial overnight stays at home during hospitalization	0.68	0.51	0.91	*0.01

* *p* < 0.05.

## DISCUSSION

In this study, we retrospectively evaluated the factors associated with treatment failure in inpatients with schizophrenia spectrum disorder and divided them into treatment during hospitalization and social environmental adjustment. We found that treatment failure within 1 year of discharge was 23.4% for inpatients with schizophrenia spectrum disorder. A previous study indicated that the readmission rate within 1 year of discharge was 38.9% for schizophrenia,^2^ and another stated that the treatment failure rate was 58.2% for schizophrenia spectrum disorders,^9^ which was not consistent with our findings. This may be because the present study is a single‐center study, and the treatment environment and post‐discharge social environment may differ from those of the patient groups described in the previous studies.

Our study found that patients undergoing antipsychotic polypharmacy may have high treatment failure rates. The antipsychotic polypharmacy rate was 36.9% in this study, whereas a previous study in Japan^6^ reported an antipsychotic monotherapy rate of 57.1% in patients with schizophrenia, suggesting consistency in the prescription status of antipsychotic medications. Antipsychotic polypharmacy may lead to adverse events, such as extrapyramidal symptoms, sexual dysfunction, somnolence, and lipid metabolism abnormalities,^7^, ^12^, ^13^, ^14^ which have been reported to trigger medication discontinuation in patients with schizophrenia.^15^ Nonadherence, including discontinuation of medication, is a known risk factor for relapse in schizophrenia spectrum disorders.^1^ Although this study did not directly evaluate patient adherence, antipsychotic polypharmacy may have caused these adverse events, leading to reduced adherence and treatment failure. Therefore, to prevent treatment failure in schizophrenia spectrum disorders, it is necessary to optimize pharmacotherapy centered on antipsychotic monotherapy, as outlined in the guidelines.^5^ However, this study did not distinguish between two‐drug combinations and three or more drugs for antipsychotic polypharmacy, nor did it evaluate drug combinations. Therefore, evaluating drug combinations is necessary in the future.

Furthermore, this study found no association between treatment failure and use of psychotropic drugs other than antipsychotics. Anticholinergics and hypnotics were reported as risk factors for hospitalization,^16^ while LAIs contributed to the prevention of treatment failure^4^; however, this was not observed in our study. In particular, it is interesting to note that no association with treatment failure was observed in LAIs in which adherence was ensured. This may be due to the small sample size of this study; therefore, further research including large sample sizes is needed.

We found that a trial overnight stay at home during hospitalization may contribute to the prevention of treatment failure. Trial overnight stays at home during hospitalization are a common practice in Japanese psychiatric hospitals; they involve patients spending one or two nights in their post‐discharge living environments while still hospitalized. No association between trial overnight stays at home during hospitalization and treatment failure has been previously reported, making this a novel finding. Establishing cooperative systems within the community and sharing goals about community life has been reported as part of the in‐hospital nursing care aimed at reducing readmissions for schizophrenia.^17^ Overnight stays at home during hospitalization may contribute to preventing treatment failure by promoting these in‐hospital nursing care measures. However, as this study did not conduct a specific survey on the hospital nursing care measures related to trial overnight stays at home, the above considerations remain speculative. Further research into the content of hospital nursing care for trial overnight stays at home is required.

Trial overnight stays at home during hospitalization may extend the hospitalization period, as psychiatric inpatients who received support from community care networks had longer hospital stays.^18^ Japan has more psychiatric beds than other countries,^19^ and longer psychiatric hospitalization periods have been reported,^20^ which have been indicated as factors contributing to delays in the deinstitutionalization of mental healthcare patients in Japan.^21^ However, the current psychiatric care environment in Japan may also facilitate accommodating additional trial overnight stays at home during hospitalization. The results of this study suggest that social environmental adjustments aimed at post‐discharge living, such as trial overnight stays at home during hospitalization, may contribute to preventing treatment failure. However, whether such practices can be widely adopted beyond the Japanese settings requires individual consideration based on each country's healthcare systems.

## LIMITATIONS

This study had certain limitations. First, the severity of the psychiatric symptoms was not evaluated using assessment scales. Therefore, the impact of the severity of the patient's condition at discharge on treatment failure could not be considered. Second, this study did not distinguish between the use of two antipsychotic drugs and the use of three or more, nor did it evaluate the combination of drugs used. In addition, this study did not evaluate the equivalent doses of antipsychotics, including chlorpromazine‐equivalent doses. Furthermore, it did not evaluate the proportion of patients who experienced drug‐related side effects. Therefore, there are limitations to generalizing the results to all cases of antipsychotic polypharmacy. Third, this study did not evaluate post‐discharge adherence, changes in treatment content, or sociocultural factors; therefore, the impact of these factors on treatment failure could not be considered. Fourth, this study was conducted at a single institution; therefore, generalizing the results may not be possible. Fifth, this was a retrospective study; therefore, further verification through prospective studies is required. Sixth, in this study, multiple hospitalizations of the same patient during the data collection period were treated as separate cases. Therefore, multiple registrations of the same patient could have impacted the results. Seventh, this study did not evaluate the presence of physical illnesses or other mental disorders. Therefore, further research including information on these comorbidities is necessary. Given these limitations, future large‐scale prospective studies across multiple institutions should include severity assessments using evaluation scales, drug combination evaluations, and consideration of confounding factors related to post‐discharge treatment content and social and environmental factors.

## CONCLUSION

Treatment failure was 23.4% within 1 year of discharge in patients with schizophrenia spectrum disorders. Antipsychotic polypharmacy at discharge was associated with treatment failure. Additionally, trial overnight stays at home during hospitalization potentially contributed to the prevention of treatment failure. To prevent treatment failure in patients with schizophrenia spectrum disorder, it is necessary to optimize pharmacotherapy and implement social and environmental adjustments with a focus on the post‐discharge period.

## AUTHOR CONTRIBUTIONS

Yoshitaka Saito collected and analyzed the data and wrote the first draft of the manuscript. Hiroyuki Muraoka and Satoru Oishi designed the study, interpreted the data, and wrote the manuscript. Takayuki Yokoyama, Katsuichiro Shimizu, Yukihiro Tanno, Nagomi Kida, Taiyo Nishikawa, Shin Miyake, Yukio Kenmotsu, Kosei Kamimura, Hirohito Kato, Yuka Noguchi, Seita Tsuchida, Ryutaro Suzuki, Yuki Yoshimura, Shoko MiuraShunsuke Kamitani, Takaaki Hirooka, and Enami Sawayama recruited participants and performed data collection and interpretation. Ken Inada supervised the entire project and performed data collection, design, analysis, and interpretation. All authors have approved the final version of the manuscript and agreed to be accountable for all aspects of the study.

## CONFLICT OF INTEREST STATEMENT

Hiroyuki Muraoka has received personal fees from Eisai, Janssen, Lundbeck Japan, Takeda Pharmaceutical Company, Meiji Seika Pharma, Mochida, MSD, Otsuka, Pfizer, Viatris, and Sumitomo Pharma over the last 3 years. Satoru Oishi received personal fees from Otsuka Pharmaceutical Co., Ltd.; Meiji Seika Pharma Co., Ltd.; Astellas Pharma Inc.; Towa Pharmaceutical Co., Ltd.; Yoshitomi Pharmaceutical Industries, Ltd.; Sumitomo Pharma Co., Ltd.; Lundbeck, Japan K.K.; and Viatris Pharmaceuticals Japan Inc. Takaaki Hirooka has received personal fees from Eisai Co., Ltd., Janssen Pharmaceutical K.K., Sumitomo Pharma Co., Ltd., Takeda Pharmaceutical Company Limited, Yoshitomi Pharmaceutical Industries, Ltd., and Viatris Pharmaceuticals Japan Inc. in the last 3 years. Ken Inada received personal fees from Daiichi Sankyo, Eisai, Eli Lilly, Janssen, Lundbeck Japan, Meiji Seika Pharma, Mitsubishi Tanabe Pharma, Mochida, MSD, Nipro, Novartis, Otsuka, Pfizer, Shionogi, Sumitomo Pharma, Yoshitomiyakuhin, and Viatris, and he received research grant support from Mochida and Sumitomo Pharma. The other authors declare that they have no competing interests.

## ETHICS APPROVAL STATEMENT

This study was approved by the Ethics Committee of Kitasato University Hospital (approval no. B22‐239, date of approval: June 1, 2023) and conducted in accordance with the ethical standards of the Declaration of Helsinki. Because this was a retrospective observational study using existing medical information, the need for signed informed consent from patients was waived by the Kitasato University Hospital Ethics Committee. Patients were informed about the purpose and procedures of the study and were given the option to opt out or decline participation.

## PATIENT CONSENT STATEMENT

Not applicable.

## CLINICAL TRIAL REGISTRATION

Not applicable.

## Data Availability

The data that support the findings of this study are available on request from the corresponding author. The data are not publicly available due to privacy or ethical restrictions.
